# Recognition of a glycosylation substrate by the O-GlcNAc transferase TPR repeats

**DOI:** 10.1098/rsob.170078

**Published:** 2017-06-28

**Authors:** Karim Rafie, Olawale Raimi, Andrew T. Ferenbach, Vladimir S. Borodkin, Vaibhav Kapuria, Daan M. F. van Aalten

**Affiliations:** 1 Centre for Gene Regulation and Expression, School of Life Sciences, University of Dundee, Dundee, UK; 2 Center for Integrative Genomics, University of Lausanne1015, Switzerland

**Keywords:** glycosylation, signalling, O-GlcNAc, O-GlcNAc transferase, substrate recognition

## Abstract

O-linked *N*-acetylglucosamine (O-GlcNAc) is an essential and dynamic post-translational modification found on hundreds of nucleocytoplasmic proteins in metazoa. Although a single enzyme, O-GlcNAc transferase (OGT), generates the entire cytosolic O-GlcNAc proteome, it is not understood how it recognizes its protein substrates, targeting only a fraction of serines/threonines in the metazoan proteome for glycosylation. We describe a trapped complex of human OGT with the C-terminal domain of TAB1, a key innate immunity-signalling O-GlcNAc protein, revealing extensive interactions with the tetratricopeptide repeats of OGT. Confirmed by mutagenesis, this interaction suggests that glycosylation substrate specificity is achieved by recognition of a degenerate sequon in the active site combined with an extended conformation C-terminal of the O-GlcNAc target site.

## 1. Introduction

The attachment of a single β-*N*-acetylglucosamine (O-GlcNAc) sugar onto serine and threonine residues of nucleocytoplasmic proteins is a dynamic and abundant post-translational modification found in higher eukaryotes [1–3]. Remarkably, this modification is regulated by only two antagonistic enzymes: the O-GlcNAc transferase (OGT), which transfers the GlcNAc moiety onto acceptor residues from the donor sugar nucleotide UDP-GlcNAc, and the O-GlcNAc hydrolase (OGA), which removes it. To date more than 1000 O-GlcNAc proteins have been identified by mass spectrometry [4–10]. These proteins cover a wide range of cellular processes such as transcription and translation [11–13], trafficking and localization [14,15], as well as cell cycle progression [16–19]. However, it remains unclear how a single OGT enzyme is able to specifically recognize a limited number of serines/threonines on such a large number of substrates.

OGT is a multi-domain protein with a catalytic core at the C-terminus and 13 tetratricopeptide (TPR) repeats at the N-terminus, making up about half of the enzyme. Early experiments suggested that the TPR domain is involved in substrate recognition and/or protein–protein interactions [20–27]. The structure of the isolated OGT TPR domain revealed topological similarity to other helical repeat proteins and led to speculation that this domain might bind substrates in an extended conformation [20]. The first structural insights into the OGT catalytic domain came from an OGT orthologue in the bacterium *Xanthomonas campestris* [28,29]. This structure revealed that the sugar donor binding site is made up of the two lobes of the glycosyl transferase B (GT-B) fold, tightly fused to the superhelical TPR domain [28]. The subsequent structure of human OGT [30] revealed a very similar fold with the addition of an intervening domain of unknown function between the two catalytic lobes [30]. The structure suggested an ordered bi-bi mechanism of substrate binding, in which UDP-GlcNAc binds before the acceptor substrate [30]. Initial structural studies exploring Michaelis/substrate complexes with short acceptor peptides have revealed limited substrate interactions with the enzyme [31,32]. More recently, Pathak *et al.* investigated the common binding modes of acceptor peptides to OGT [33]. Starting from a peptide library, they identified preference for certain acceptor peptide sequences, leading to definition of a degenerate sequon of OGT peptide substrates ([TS][PT][VT][S/T][RLV][ASY]). Crystal structures of complexes of OGT with some of these peptides revealed that OGT binds all the acceptor peptides studied so far in an extended conformation with similar conformation of the residues in the −3 to +2 position around the acceptor serine/threonine. Although the C-termini of these peptides point towards the TPR domain, these structural data do not explain how OGT recognizes larger protein substrates for glycosylation. The short sequence patterns alone are not sufficient to accurately predict the O-GlcNAc proteome, suggesting other mechanisms contribute to substrate recognition.

A clue to how this might work came from the unusual OGT substrate host cell factor 1 (HCF1). HCF1 is a ubiquitously expressed chromatin-associated protein and a major transcriptional co-regulator involved in numerous cellular processes such as cell cycle progression (reviewed in [34]), which has also been shown to be heavily O-GlcNAcylated [35]. HCF1 is initially expressed as an approximately 210 kDa protein that is activated by limited proteolysis (protease maturation) within the proteolytic processing domain (PPD), consisting of multiple 20-residue repeats [36,37]. Strikingly, in 2011 it was discovered that OGT not only glycosylates HCF1 but is also needed for its proteolytic maturation [13,35]. A depletion of OGT leads to an accumulation of full-length HCF1 protein and the PPD is proteolytically cleaved by OGT via an unusual glycosylated glutamate intermediate [24,35,38]. A recent structural study of a short PPD (HCF1_PRO_) repeat in complex with OGT revealed that part of the substrate bound in extended conformation in the TPR repeats [24]. OGT was shown to form an extensive array of polar interactions with the backbone of the HCF1_PRO_ repeat peptide, as well as specific side chain interactions that were demonstrated to be essential for HCF1_PRO_ binding [24]. However, it is as yet not clear if this binding mode also extends to OGT glycosylation substrates.

A well-characterized OGT glycosylation substrate is the TGFβ-activated kinase 1 (TAK1) binding protein 1 (TAB1), a pseudophosphatase involved in the TGFβ-mediated inflammatory signalling pathway and found to be an essential activator of TAK1 [39,40]. The structure of the TAB1 N-terminal pseudophosphatase domain has been reported and revealed similarity to the PPM family of protein Ser/Thr protein phosphatases [41]. Previous studies have shown that phosphorylation at a C-terminal region of TAB1 regulates TAK1 activity [42–44]. We have recently discovered that TAB1 is dynamically O-GlcNAcylated at Ser395 in the C-terminal domain [45]. This glycosylation appears to be required for full activity of TAK1 and activation of downstream transcription and secretion of pro-inflammatory cytokines. Here, we exploit a novel approach to covalently trap OGT-substrate complexes to explore how OGT recognizes glycosylation substrates through its TPR domain. The structure of the OGT in complex with the TAB1 C-terminal domain combined with mutagenesis studies reveals that OGT recognizes the TAB1 substrate, and by extension a group of glycosylation substrates with similar disordered regions, through extensive essential interactions with the TPR repeats.

## 2. Results and discussion

### 2.1. The TAB1 O-GlcNAc site resides in a disordered region with similarity to other OGT targets

**Figure 1.**
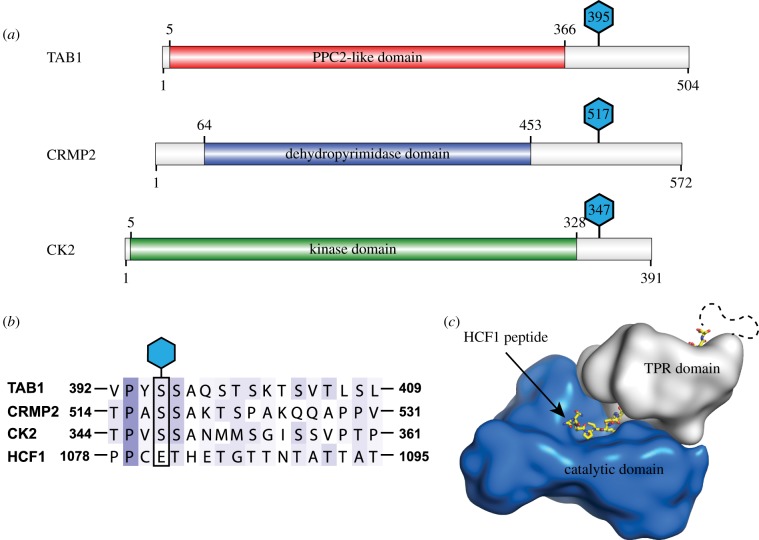
O-GlcNAc sites for a subset of proteins are located in a C-terminal disordered domain. (*a*) Cartoon depicting the domain structure and location of the glycosylation sites of the three OGT substrates TAB1, CRMP2 and CK2. The O-GlcNAc sites are depicted as blue hexagons. (*b*) Sequence alignment of the O-GlcNAc sites of TAB1, CRMP2 and CK2 as well as an HCF1_PRO_ repeat. The O-GlcNAc sites for TAB1, CRMP2 and CK2, as well as the corresponding glutamate for the HCF1_PRO_ repeat are highlighted with a black box. The sequence alignment shows the similarity between TAB1, CK2, CRMP2 and HCF1 surrounding the O-GlcNAc sites. (*c*) Schematic of OGT with a bound HCF1 peptide. The catalytic domain is shown in blue; the tetratricot-peptide repeat domain is shown in grey and the HCF1 peptide as yellow sticks (PDBID 4N3B).

**Figure 2.**
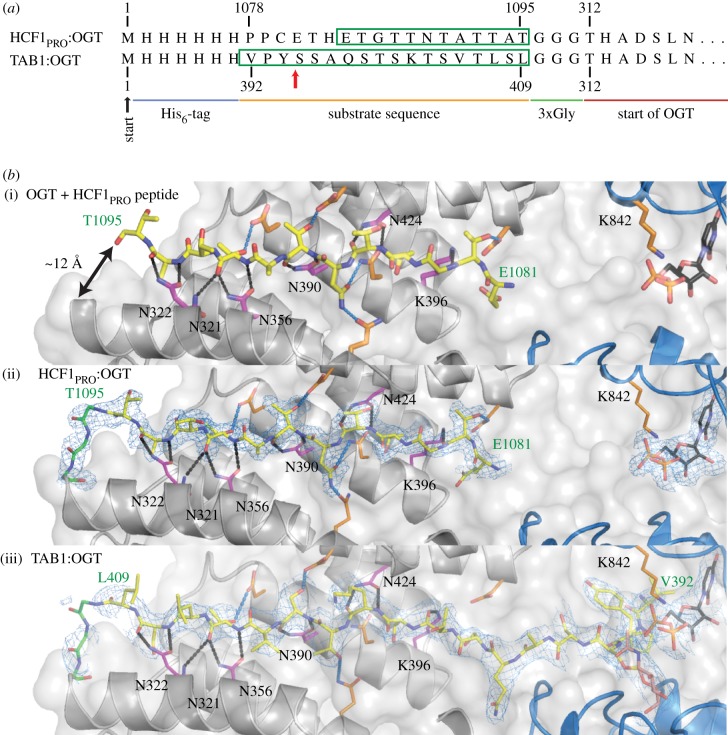
Design and structures of OGT:substrate fusion proteins. (*a*) Partial sequence of the fusion proteins showing the His_6_-tag, the sequence of the substrate peptide, the 3xGly linker and the start of the OGT protein. Construct boundaries are indicated. The reported O-GlcNAc site Ser395 on TAB1 [45] is highlighted with a red arrow. Visible residues in the respective fusion construct structures are highlighted by a green box. (*b*) Three panels showing the structures of the free HCF1_PRO_ repeat (PDBID 4N39) bound to OGT (i), the fusion protein HCF1_PRO_:OGT (ii) and the fusion protein TAB1:OGT (iii). OGT is shown in cartoon representation with the TPR and catalytic domains in grey and blue, respectively. The substrate peptides, UDP and the GlcNAc residues are shown as yellow, black and pink sticks, respectively. The 3xGly linker in the fusion constructs is shown as green sticks. OGT residues interacting with the backbone of the substrate peptide are shown as magenta sticks and residues interacting with side chains are shown as orange sticks. The catalytically important K842 residue is shown as orange sticks. The green labels highlight the start and end residues of the substrate part of the fusion constructs. The distance from the N-terminus of OGT and the C-terminus of the free HCF1_PRO_ peptide (top) is shown as a black double-headed arrow. Polar interactions between OGT and the backbone and side chains of substrate peptides are shown as black and blue dashed lines, respectively. The *F*_o_−*F*_c_ map for the fusion constructs HCF1_PRO_:OGT and TAB1:OGT are shown as light-blue mesh contoured to 2.5 σ.

The O-GlcNAcylation sites on the OGT substrates TAB1 [45], collapsin response mediator 2 protein (CRMP2) [9] and casein kinase 2 (CK2) [46] are located in disordered regions close to the C-terminus (figure 1*a*). Although short peptides derived from these sites can be co-crystallized with OGT [31–33], we have been unsuccessful in using this approach with longer sequences/intact proteins to explore the role of the OGT TPR domain in substrate recognition. Aligning the sequences around the O-GlcNAc sites reveals similarities near the site of modification (figure 1*b*). Remarkably, this is also similar to the proteolytic cleavage site of a HCF1_PRO_ repeat, with the major difference being a glutamate at the acceptor position (figure 1*b*). Mutating this glutamate to a serine is sufficient to change the peptide from a proteolytic to a glycosylation substrate [24]. In the OGT:HCF1_PRO_ structure [24], the peptide substrate spans the whole length of the TPR domain (figure 2*b*). The peptide interacts with the TPRs through some of its side chains, but intriguingly five regularly spaced asparagine side chains in OGT form hydrogen bonds with the HCF1_PRO_ peptide backbone in a sequence-independent manner (figure 2*b*). We noted the fortuitous proximity of the HCF1_PRO_ C-terminus to the OGT N-terminus (Cα_HCF1–1340_-Cα_OGT313_ approx. 12 Å; figure 2*b*), and wondered whether this would enable the direct tethering of substrates to OGT via a fusion linker to allow us to explore OGT-glycosylation substrate complexes.

### 2.2. A linear fusion of OGT and HCF1_PRO_ reproduces the HCF1_PRO_ binding mode

To explore whether a fusion of the C-terminus of a peptide substrate to the N-terminus of a truncated OGT (312–1031) would generate physiological OGT-substrate complexes, we explored this approach first with HCF1_PRO_. A construct was designed where an 18-mer HCF1_PRO_ repeat peptide (PPCETHETGTTNTATTAT) was fused to the N-terminal Thr315 of OGT via a three glycine (3xGly) linker (figure 2*a*). The fusion construct was overexpressed as a His_6_-tagged protein in *E. coli*, purified and crystallized. Well-diffracting protein crystals were obtained and synchrotron data were collected to 1.9 Å (electronic supplementary material, table S1). Molecular replacement and subsequent refinement revealed continuous unbiased |*F*_o_|–|*F*_c_| density for both the HCF1_PRO_ peptide and the 3xGly linker (figure 2*b*). Encouragingly, the conformation of the HCF1_PRO_ peptide in the fusion protein was nearly identical to that observed in the previously published OGT-HCF1 peptide complex [24] (RMSD on Cα atoms = 0.2 Å). Thus, just as with the free peptide, the tethered HCF1_PRO_ peptide backbone binds the OGT TPR domain in an extended conformation, interacting with residues lining the concave surface of the TPR superhelix (figure 2*b*). Therefore, a linear fusion of OGT and HCF1_PRO_ reproduces the HCF1_PRO_ binding mode.

### 2.3. A linear TAB1:OGT fusion suggests that TAB1 makes extensive interactions with the OGT TPRs

**Figure 3.**
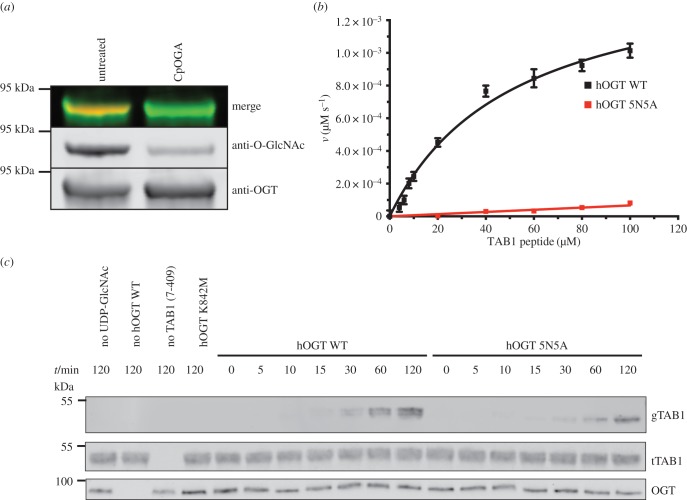
Activity of wild-type and mutant OGT on peptide and protein substrates. (*a*) Western blot analysis of CpOGA treated purified TAB1:OGT fusion protein. Briefly, 0.5 µg of TAB1:OGT was incubated in the presence and absence of approximately 10 µg ml^−1^ CpOGA for 30 min at 37°C. Reactions were stopped by addition of LDS-loading buffer and boiling at 95°C. (*b*) Graph showing the steady-state kinetics to determine the *K*_M_ of the TAB1 peptide (KKPVSVPYSSAQSTSKTSVTLSL). Briefly, 50 nM hOGT(WT/5N5A) was pre-incubated with varying concentrations of TAB1 peptide before starting the reaction by adding UDP-GlcNAc to a final concentration of 50 µM. The reaction was stopped before 10% of substrate was converted by addition of detection reagent in 50% MeOH. *K*_M_ (WT) = 42 ± 7 µM, *K*_M_ (5N5A) = N.D. (*c*) Western blot analysis of *in vitro* glycosylation reactions of TAB1 with OGT (WT/mutants). Briefly, 10 µM TAB1 protein was incubated with 50 nM hOGT in 100 µl 0.1 M Tris-HCl pH 7.4, 0.15 M NaCl, 0.5 mM TCEP buffer. Reactions were started by the addition of UDP-GlcNAc to a final concentration of 100 µM and incubated at 25°C. Samples were taken at indicated time points and reactions stopped by boiling for 5 min at 95°C in LDS-loading buffer.

We next explored the OGT-substrate fusion approach as a means of trapping complexes of OGT with TAB1. We generated a TAB1:OGT fusion construct matching the HCF1_PRO_:OGT fusion, using an 18-mer TAB1 peptide derived from the S395 glycosylation site (VPY**S**SAQSTSKTSVTLSL; figure 2*a*). The chimaeric protein was overexpressed as a His_6_-fusion construct in *E. coli* and purified as described for the HCF1_PRO_:OGT fusion protein (figure 2*a*). We were able to generate crystals of the TAB1:OGT fusion protein, solve the structure by molecular replacement and refine the complex against 2.5 Å synchrotron diffraction data to *R*_work_/*R*_free_ = 0.22/0.25 (electronic supplementary material, table S1). The unbiased |*F*_o_|–|*F*_c_| density allowed unambiguous building of the linker and peptide (figure 2*b*). The first eight amino acids of the TAB1 peptide (VPY**S**SAQS), covering the glycosylation site, were found in a similar conformation in the active site to the free TAB1 peptide in complex with OGT reported previously [31] (figure 2*b*, RMSD on Cαs = 1.4 Å). The electron density revealed Ser395 to be glycosylated as a result of self-glycosylation during expression in *E. coli*, which was confirmed by western blot analysis (figure 3*a*). The sugar occupies the same position as observed in a complex with a short synthetic TAB1 glycopeptide [31] (maximum atomic shift = 0.1 Å**)**. Intriguingly, there appears to be some extra electron density near Ser396 and Ser399 suggestive of additional glycosylation sites (electronic supplementary material, figure S1) that could be an artefact of the very high (local) concentrations of the fused substrate peptide, or glycosylation occurring in *trans* as a result of the high protein concentrations (approx. 10 mg ml^−1^) used in the crystallization experiments. In the TAB1:OGT fusion structure, the TAB1 peptide forms two side-chain-mediated interactions (Ser404/Thr406) with the TPR domain of OGT (Asp386/Asp420) (figure 2*b*). These are remarkably similar to the interactions between the same OGT residues and Thr1090 and Thr1092 of the HCF1_PRO_ repeat (figure 2*b*). Similarly, the interactions between the TAB1/HCF1 peptide backbones and the five regularly spaced OGT TPR asparagines are conserved (figure 2*b*). Furthermore, the overall conformations of the TAB1 and HCF1 peptides in the respective fusion constructs is similar (RMSD on Cαs = 1.3 Å). Thus, a linear TAB1:OGT fusion suggests that the TAB1 OGT substrate makes extensive interactions with the OGT TPRs.

### 2.4. Interactions with the OGT TPRs contribute to TAB1 O-GlcNAcylation

Although the similarity to the HCF1 peptide binding mode and the presence of glycosylation on Ser395 suggests we have trapped a physiologically relevant TAB1:OGT complex, we further tested this model by structure-guided site-directed mutagenesis in the context of truncated OGT (312–1031) and TAB1 (7–409) as separate proteins. Two types of OGT mutants were generated: a single-point mutant in the active site (K842M), known to be essential for catalytic activity [31] and a quintuple mutant where the five key asparagine residues that form the bulk of interactions in the TPR domain (Asn322, Asn325, Asn356, Asn390 and Asn424, figure 2*b*) were all mutated to alanines (from here on referred to as the 5N5A mutant). Based on the TAB1:OGT fusion protein complex, the 5N5A mutations would be expected to disrupt the binding of the C-terminal region of TAB1 to the TPR domain. Using western blot analysis, we probed OGT activity on TAB1 and blotted for O-GlcNAcylation using an O-GlcNAc Ser395 specific antibody [45]. As demonstrated previously, TAB1 is readily O-GlcNAcylated by WT OGT, whereas no glycosylation is observed with the catalytically inactive K842M mutant [31] (figure 3*c*). The 5N5A mutant shows significantly reduced activity on a free TAB1 peptide (KKPVSVPYSSAQSTSKTSVTLSL) matching the peptide used in the fusion construct (figure 3*b*), in agreement with the interactions formed by the key asparagines in the TPR domain of OGT observed in the structure (figure 2*b*). However, when using a shorter synthetic TAB1 peptide (KKPVSVPYSSAQSTS, ending just before the start of the TPR repeats), the 5N5A mutant shows the same activity levels as WT OGT (electronic supplementary material, figure S2*a*). Intriguingly, the 5N5A mutant appears to show a more modest reduction of glycosyltransferase activity (approx. 50%), as calculated by quantifying fluorescent signal from the fluorophore conjugated secondary antibodies used in the western blot analysis (electronic supplementary material, figure S2*b*), on the TAB1 (7–409) protein (figure 3*c*), suggesting that while interactions of the TAB1 C-terminus with the OGT TPRs are important, further interactions with the globular pseudophosphatase domain of TAB1 may exist. Nevertheless, interactions with the OGT TPRs contribute to TAB1 O-GlcNAcylation.

## 3. Concluding remarks

The human O-GlcNAc transferase is a multi-domain protein and is essential in metazoa [12,47,48]. However, it is still unclear how a single OGT enzyme recognizes its multitude of substrates. Previous work has proposed sequence specificity targeting −2 to +3 relative to the acceptor residue [33,49]. Previous work has also suggested the involvement of the TPR domain in substrate recognition by incrementally removing repeats from the TPR domain, resulting in a loss of activity on substrates even on a peptide level, although the molecular basis of this was as yet unclear [20–23,25–27]. Using the proteolytic OGT substrate HCF1, Lazarus *et al.* [24] revealed the involvement of multiple OGT residues on the concave surface of the TPR domain in binding side chains and backbone of the HCF1_PRO_ repeat proteolytic substrate. Here, we used a fusion approach to trap OGT-substrate complexes to investigate the role of the TPRs in recognition of glycosylation substrates. We first demonstrated that this fusion approach recapitulates the published HCF1_PRO_ peptide binding mode and then used that to reveal how the C-terminus of the OGT glycosylation substrate TAB1 is recognized by the enzyme. The TAB1 C-terminus binds in an extended conformation in the TPR domain, making extensive contacts with the concave surface through regularly spaced asparagines in OGT. An OGT mutant lacking these asparagines was deficient in glycosylation of TAB1. Interestingly, the data show a complete loss of O-GlcNAcylation of a free TAB1 C-terminal peptide, whereas activity on a TAB1 protein is more modestly reduced. These findings, coupled with recently published work on an OGT substrate sequence preference [33], suggest that OGT may bind its substrates through a combination of mechanisms. It is interesting to note that many other OGT substrates (e.g. Casein kinase II and CRMP2; figure 1*a*) also possess similarly disordered regions C-terminal of the O-GlcNAcylation site, suggesting that this may be a general mode of OGT substrate recognition. However, O-GlcNAc sites have also been reported to reside in/close to secondary structure motifs, as is the case for Histone H2B [50], p53 [51], the glucose-6-phosphate dehydrogenase G6PD [52] and SNAP-29 [53]. It is possible that a subset of substrates is O-GlcNAcylated in a co-translational fashion as proposed by recent work [54]. In this work, we have shown, using crystallography and site-directed mutagenesis, that the OGT substrate TAB1 binds the enzyme in the same way as the proteolytic substrate HCF1 [24] and that the five asparagine residues found on the concave surface of the TPR domain (Asn321, Asn322, Asn356, Asn390 and Asn424) are important for binding. Future studies could be directed at dissecting which other parts of OGT and/or substrate proteins contribute to substrate binding.

## 4. Material and methods

### 4.1. Construct design/cloning

A codon-optimized version of hOGT 313–1031, based on the boundaries described in [31], was ordered from GenScript and cloned as a *Bam*HI-*Not*I restriction fragment into a modified version of pGEX6P1 containing a 6His tag instead of a GST tag. PCR primers (6H_HCF1_GGG_hOGT_fwd GTATTCATGCATCATCACCACCATCACccgccctgcgagacccacg, 6H_HCF1_GGG_hOGT_rev CAGGTTGTTCAGGGAATCAGCATGGGTaccgccaccggtggcggtggtggcggtg; 6H_TAB1_GGG_hOGT_fwd GTATTCATGCATCATCACCACCATCACGTGCCATACTCCAGCGCCCAG and 6H_TAB1_GGG_hOGT_rev CAGGTTGTTCAGGGAATCAGCATGGGTaccgccaccAAGGGAGAGGGTCACGCTGGTC) were then designed to introduce a TAB1 or HCF1 peptide followed by a GGG linker in place of the PreScission Protease site and the first two residues of the hOGT, changing the boundaries to 315–1031. This PCR product was introduced into the existing construct by restriction free cloning [55].

Cloning of the quintuple mutants of hOGT was carried out by ordering a GeneBlock from Integrated DNA Technologies containing all five codon changes. This was then incorporated into the existing construct by restrictionless cloning based on [55] but using KOD polymerase and *Dpn*I from Fermentas and using primers OGT5N5A_F: CCTGTCCGACCCATGCTGATTC, OGT5N5A_R: CCGGAGTCTTTGTGAATCGATGC and GeneBlock OGT_5N5ACCTGTCCGACCCATGCTGATTCCCTGgcCgcCCTGGCGAACATTAAGCGTGAACAAGGCAACATTGAAGAAGCCGTCCGTCTGTATCGTAAAGCGCTGGAAGTCTTTCCGGAATTCGCGGCGGCACATAGTgcCCTGGCCTCCGTGCTGCAGCAACAGGGCAAGCTGCAGGAAGCTCTGATGCACTATAAAGAAGCGATTCGTATCTCTCCGACCTTTGCCGATGCATACAGTgcCATGGGTAATACGCTGAAAGAAATGCAAGACGTGCAGGGCGCCCTGCAATGTTATACCCGCGCAATTCAGATCAACCCGGCTTTCGCGGATGCCCATTCAgcTCTGGCATCGATTCACAAAGACTCCGG.

The pGEX6P1 TAB1 7-409 construct was generated from a larger fusion construct produced as above, then the GGG-hOGT region was erased using a method based on the QuikChange site-directed mutagenesis kit by Agilent but using KOD polymerase and *Dpn*I from Fermentas. All inserts were confirmed by DNA sequencing.

### 4.2. Expression and purification of linear fusion constructs

Both HCF1_PRO_:OGT and TAB1:OGT fusion constructs were recombinantly expressed as His_6_-tagged proteins in *E. coli* BL21 (DE3) pLysS. Cultures were grown in LB media, supplemented with ampicillin, until an OD_600_ of approximately 0.6 was reached. Expression was induced with 250 µM IPTG for 18 h at 16°C. Cells were harvested by centrifugation at 4800*g* in a J6-MI centrifuge (Beckman Coulter). The pellet was resuspended in lysis buffer (25 mM Tris, 150 mM NaCl, 0.5 mM TCEP and 30 mM imidazole pH 8.5, containing approx. 0.1 mg ml^−1^ lysozyme, 0.1 µg ml^−1^ DNAse, 1 mM benzamidine, 0.2 mM PMSF and 5 μM leupeptin). Cell lysate was spun down at 20 000*g* for 10 min in an Avanti J-25 centrifuge (Beckmann). The supernatant was incubated with Nickel-NTA resin for 2 h at 4°C. The beads were isolated by passing through a column and washed extensively with lysis buffer. Bound protein was eluted from the beads with lysis buffer containing 200 mM imidazole. The eluent was diluted to 25 mM NaCl in Tris–HCl pH 8.5 and purified further by anion-exchange chromatography using a HiTrap QFF 5 ml column (GE Healthcare). Fractions corresponding to the size of the fusion protein were pooled, concentrated to less than 2 ml and loaded onto a SuperDex 200 gel filtration column (GE Healthcare). Purity was checked by subjecting the fractions to SDS-PAGE analysis and pure fractions were pooled and buffered exchanged into a 50 mM Tris–HCl pH 8.5, 25 mM NaCl and 0.5 mM TCEP.

### 4.3. Crystallization and structure solution

The fusion constructs were crystallized at a protein concentration of approximately 10 mg ml^−1^ in the presence of 5 mM UDP. Sitting drop vapour diffusion experiments were performed by combining 0.5 µl of protein with 0.5 µl mother liquor. Crystals grew in 2–3 days in 3.5 M sodium formate, 0.1 M Tris pH 8.5 for TAB1-OGT fusion and 1.3 M ammonium tartrate dibasic, 0.1 M Tris pH 8.5 for HCF1_PRO_:OGT. Crystals were cryoprotected using 10% glycerol in mother liquor and 2.5 M lithium sulfate for TAB1:OGT and HCF1_PRO_:OGT, respectively. Data were collected at the European Synchrotron Radiation Facility (ESRF) beam line ID29 and were autoprocessed with XDS [56,57]. Structures were solved by molecular replacement using Molrep [58] and chain A of PDB 3PE4 [30] as search model. Crystals belong to space groups *P*6_1_22 (TAB1:OGT) and *P*6_2_22 (HCF1_PRO_:OGT) and have one molecule per asymmetric unit. A ligand topology for UDP was created with PRODRG [59]. The structures were fully refined using iterative cycles of Refmac5 [60] and manual building with COOT [61]. Data collection and refinement statistics can be found in the electronic supplementary material, table S1.

### 4.4. *In vitro* TAB1 glycosylation assay

For glycosylation assays, TAB1 and hOGT (WT and mutants) were expressed and purified as described previously [30,31,33,41]. Ten mircomolar of TAB1 was incubated with 50 nM hOGT (WT or mutants) in TBS reaction buffer (0.1 M Tris–HCl pH 7.4, 150 mM NaCl) supplemented with 0.5 mM TCEP and 0.1 mg ml^−1^ BSA. The reaction was started by adding UDP-GlcNAc to a final concentration of 100 µM and incubating the reaction mixtures at 25°C. Ten microlitres of sample mixtures was taken at indicated times and mixed with 4x LDS sample loading buffer to a final volume of 50 µl and boiled at 95°C for 5 min. Proteins were resolved using precast SDS-PAGE gels (NuPAGE 4–12% Bis-Tris gels, Invitrogen) and blotted onto nitrocellulose membranes (GE Healthcare). The primary antibodies were used at the following concentrations: Anti*-*TAB1-O-GlcNAc (1 : 1000 [45]), anti-TAB1 (1 : 1000, Division of Signal Transduction and Translation, University of Dundee) and anti-OGT (1 : 2000, DM17, Sigma-Aldrich, Cat#: O6264). Li-Cor secondary antibodies (IRDye 680 Donkey anti-rabbit and IRDye 800 Donkey anti-rabbit, anti-sheep) were used at dilutions of 1 : 10 000. Blots were imaged using the Li-Cor Odyssey infrared imaging system (Li-Cor, Lincoln, NE). Quantification of the O-GlcNAc specific signal (gTAB1) was performed using imageStudioLite (Li-Core) and normalized to total OGT (tOGT) and total TAB1 (tTAB1) signal. Data were plotted with GraphPad Prism 7.

### 4.5. Steady-state kinetics

hOGT activity was determined in reactions containing 50 nM of either WT or 5N5A His6-hOGT (312–1031), 50 mM Tris–HCl pH 7.4, 0.1 mg ml^−1^ BSA, 10 µM sodium dithionate and varying concentrations of the TAB1 peptide KKPVSVPYSSAQSTSKTSVTLSL or at a fixed concentration of 10 µM of the TAB1 peptide KKPVSVPYSSAQSTS, in a total volume of 100 µl. Reaction mixtures were preincubated for 15 min before initiating the reaction by adding UDP-GlcNAc to a final concentration of 50 µM. Reactions were incubated for 30 min at 21°C before addition of 200 µl of 75 µM pyrocatechol violet/15 µM fluorophore, DP-sensitive xanthene-based Zn(II) compound [33,62,63], in 25 mM HEPES pH 7.4, 10 mM NaCl, 50% (v/v) MeOH. UDP formation was detected on a Gemini EM fluorescent Microplate reader (Molecular Devices) using excitation and emission wavelengths of 485 nm and 530 nm, respectively. Turnover did not exceed 10% for either substrate. Data are presented as average of three measurements, with error bars showing s.e.m. Data were analysed with GraphPad Prism 7.

### 4.6. Western blot analysis of purified TAB1:OGT

Samples of purified TAB1:OGT fusion protein were incubated for 30 min at 37°C in the presence and absence of approximately 10 µg ml^−1^ CpOGA, a promiscuous bacterial O-GlcNAc hydrolase [64]. Samples were supplemented with 4x LDS-loading buffer and boiled for 5 min at 95°C. A total of 0.5 µg of each untreated and treated TAB1:OGT fusion protein were subjected to SDS-PAGE analysis and transferred onto a nitrocellulose membrane (GE Healthcare), using a wet-transfer system (Invitrogen). The membrane was blocked in 5% BSA for 30 min at 21°C before incubating with anti-O-GlcNAc AB (RL2, 1 : 1000, Abcam, catalogue no. ab2739) and anti-OGT AB (1 : 2000, Abcam, catalogue no. 177941). Li-Cor secondary antibodies IRDye 680 Donkey anti-mouse (anti-O-GlcNAc) and IRDye 800 Donkey anti-rabbit (anti-OGT) were used at dilutions of 1 : 10 000. Blots were imaged using the Li-Cor Odyssey infrared imaging system (Li-Cor, Lincoln, NE).

### 4.7. Peptide synthesis

Microwave-assisted solid-phase peptide synthesis was performed with CEM Liberty automated peptide synthesizer on Rink amide MBHA resin (Novabiochem) using standard Fmoc chemistry protocols. The peptide was cleaved from the resin and deprotected with i-Pr_3_SiH-H_2_O-TFA 2.5 : 5 : 92.5 mixture for 2 h. The crude peptide was obtained after dilution of the cleavage mixture with diethyl ether and centrifugation. It was finally purified by reverse-phase HPLC at C18 Waters Xbridge OBD 5 µm 19 × 100 column in a linear gradient of buffer B (acetonitrile−0.1% trifluoroacetic acid) in buffer A (water−0.1% trifluoroacetic acid) 5–40% in 5 min, flow rate 20 ml min^−1^. Appropriate fractions were pooled and freeze-dried to provide the target compound as fluffy solid.

## 5. Accession codes

X-ray diffraction data and refined structures have been deposited in the Protein Data Bank under accession codes 5LWV (HCF1_PRO_:OGT) and 5LVV (TAB1:OGT).

## Supplementary Material

Figure S1

## Supplementary Material

Figure S2

## Supplementary Material

Table S1

## Data accessibility

This article has no additional data.
