# Supplementary figures and images for: Recognition of a glycosylation substrate by the O-GlcNAc transferase TPR repeats
Source: Open Biol. 2017 Jun 28;7(6):170078. doi: 10.1098/rsob.170078 (PMC5493779; doi:10.1098/rsob.170078)

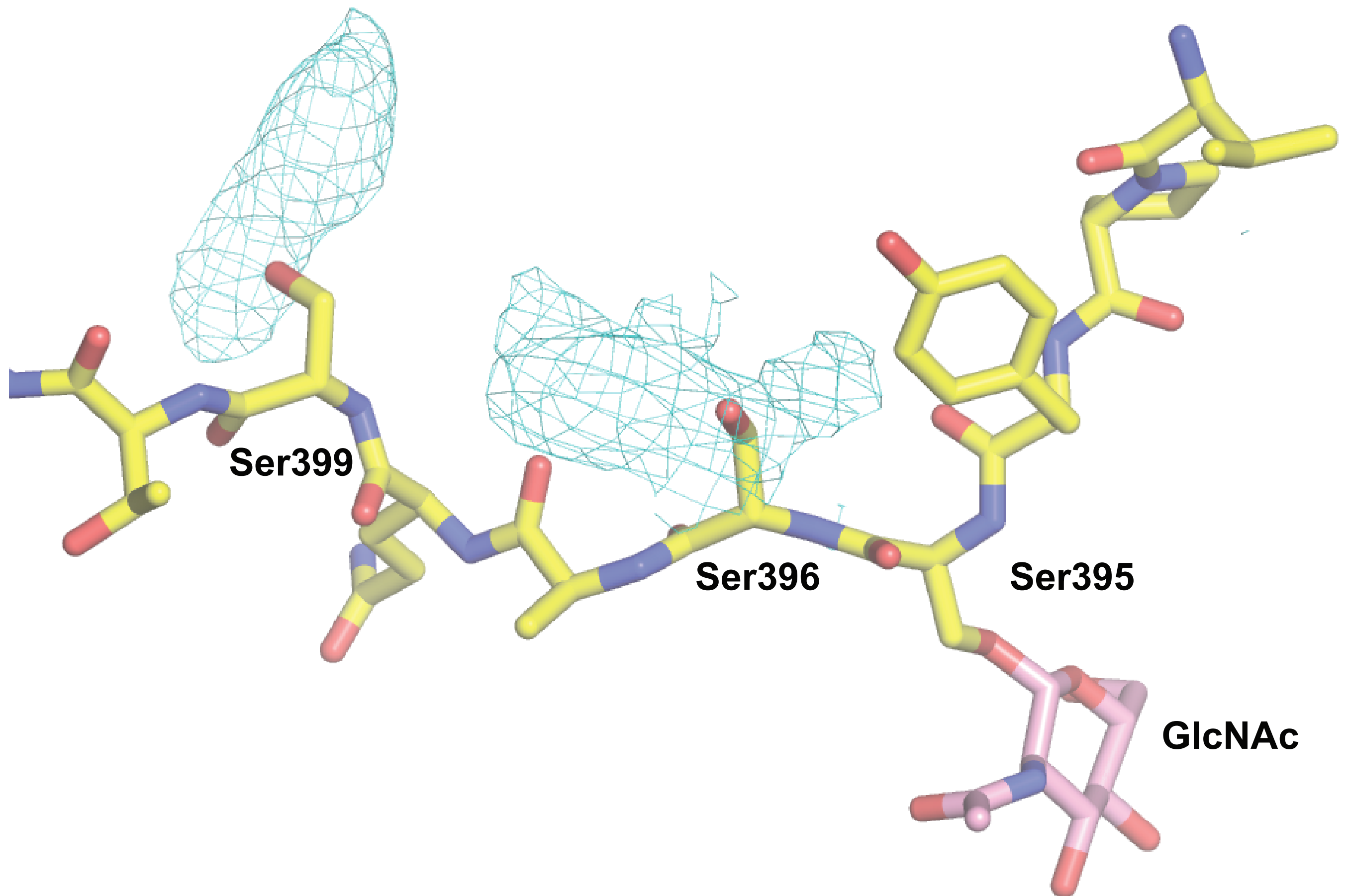

Supplement: Figure S1 [file rsob170078supp1.pdf]

A

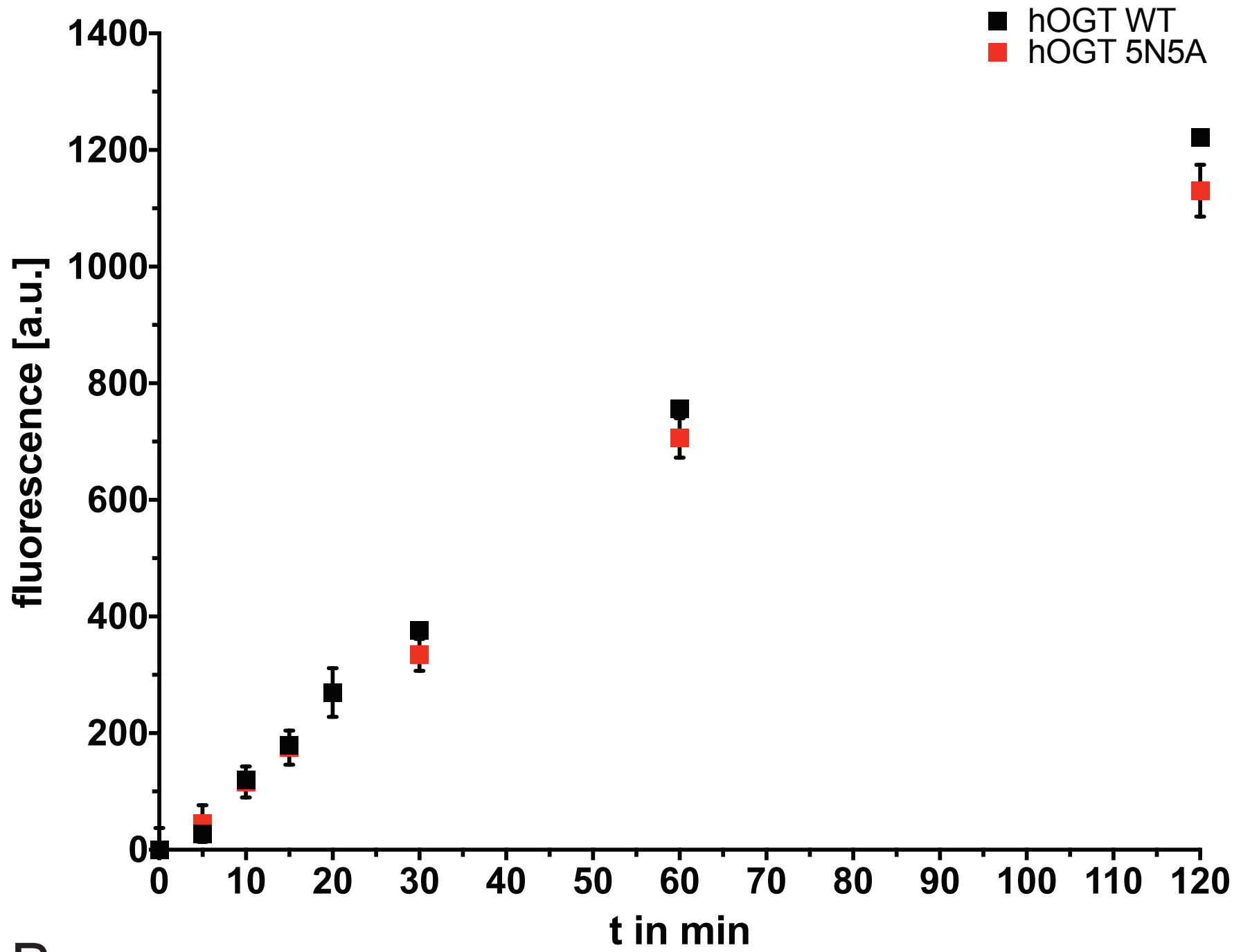

B

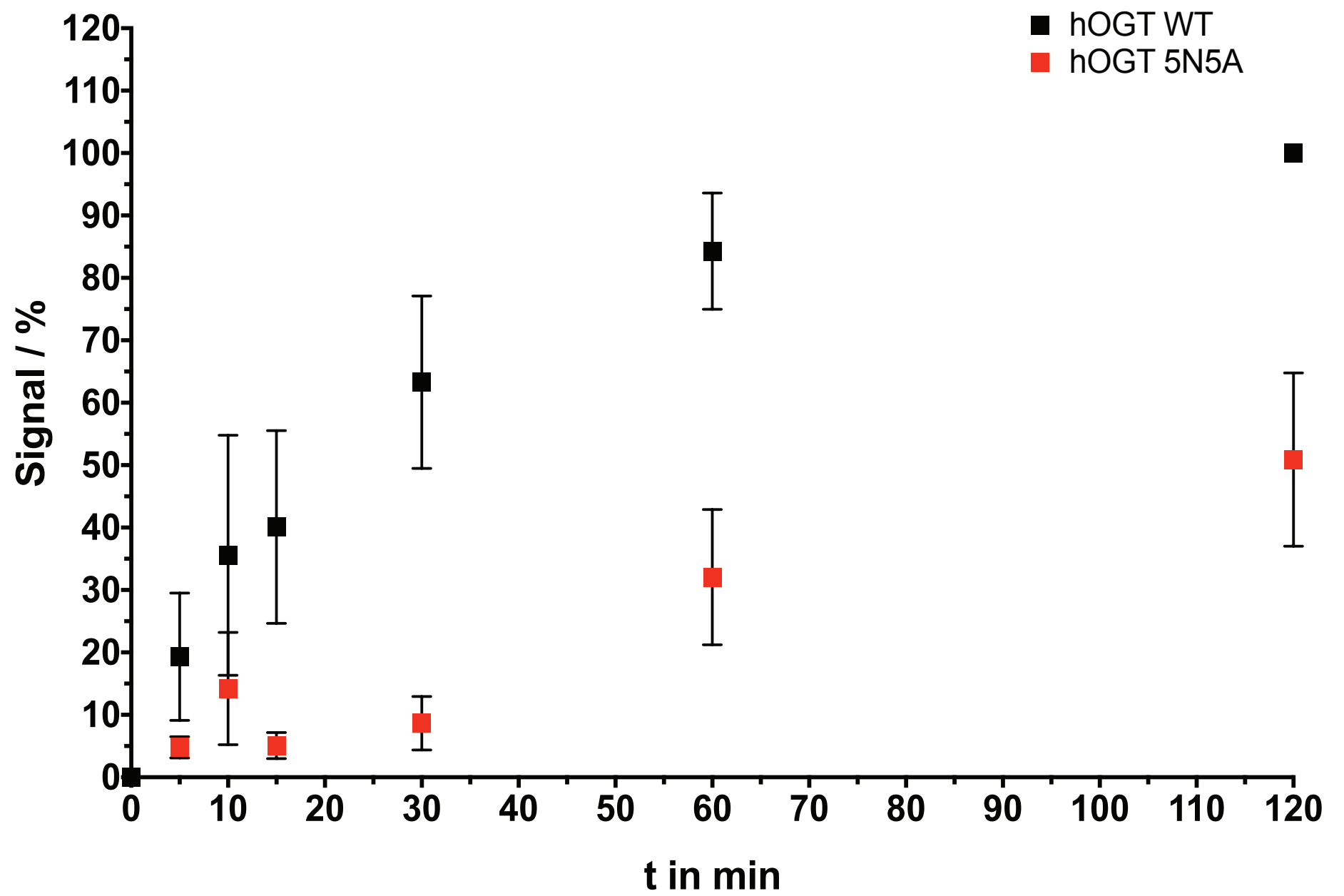

Supplement: Figure S2 [file rsob170078supp2.pdf]
