# Supplementary material for: Recognition of a glycosylation substrate by the O-GlcNAc transferase TPR repeats
Source: Open Biol. 2017 Jun 28;7(6):170078. doi: 10.1098/rsob.170078 (PMC5493779; doi:10.1098/rsob.170078)
Supplement: Table S1 [file rsob170078supp3.pdf]

Table S1. Data collection and refinement statistics.

	$HCF1_{PRO}$:OGT	TAB1:OGT
data collection		
beamline, wavelength	ID29, 0.976 Å	ID29, 0.976 Å
space group	*P6222*	*P6122*
cell dimensions (Å)	$a = b$ = 168.1, c = 162.5	$a = b$ = 98.5, c = 367.2
cell dimensions (°)	$\alpha = \beta$ = 90.0, γ = 120.0	$\alpha = \beta$ = 90.0, γ = 120.0
resolution (Å)	48.5–1.90 (1.97–1.90)	49.73–2.54 (2.63–2.54)
R_{merge}	0.147 (1.95)	0.134 (1.95)
$I/\sigma I$	14.4 (1.7)	16.3 (1.8)
$CC_{1/2}$	1.00 (0.68)	1.00 (0.83)
R_{meas}	0.154 (2.007)	0.141 (2.044)
R_{pim}	0.047 (0.628)	0.044 (0.621)
completeness (%)	100 (99.7)	99.8 (99.8)
redundancy	20 (20.2)	19.2 (20.3)
refinement (Å)	48.5–1.90	49.73–2.54
no. total reflections	101 030	36 043
no. unique reflections	9926	34 136
R_{work}, R_{free}	0.174/0.199	0.193/0.245
B-factor average		
protein	29.9	72.6
peptide	33.6	65.9
RMSD		
bond lengths (Å)	0.021	0.014
bond angles (°)	2.0	1.8
PDB ID	5LWV	5LVV
